# The Squandering of Posthumous Gifts to Charity

**Published:** 1890-03-29

**Authors:** 


### Passing Topics.

### THE SQUANDERING OF POSTHUMOUS GIFTS TO CHARITY.

Signs are not wanting that another considerable fund left for distribution "at the discretion" of executors is to be scattered in a multitude of trivial benefactions. Day by day, one and another institution is reported in the columns of the newspapers to have obtained a grant of £50, £100, or it may be £200, preparatory, no doubt, to the early publication of a stupendous list of recipients, in which the executors will demonstrate nothing so conclusively as the incontinence of their methods and the ease with which they have been led into manifold indiscretions.

Announcements of similar purport appear at not infrequent intervals, and we believe that during a period of, say, fifteen years, a sum approaching to two millions of money has been disposed of in this way among metropolitan charities, good, bad, and indifferent. Of all this vast amount how much has been saved from annihilation? It is questionable if any of the two millions poured through so fine a sieve has failed to evaporate in the process. The grand gifts which might have proved sources of perennial nourishment, or might have been applied to the promotion of definite and lasting objects have vanished, and scarcely one institution has been made stronger, more useful, or more independent.

Of course, the desire to benefit a very large number of the appealing charities springs from irreproachable motives, though the temptation to dissolve a great opportunity into a multitude of petty incidents seems one to be resisted. Equally, of course, institutions are thankful for the smallest mercies, and with the hunger and improvidence of their order swallow to-day's dole without a thought of the wants of to-morrow. But why should the rules of prudence which commonly guide the actions of business men be forgotten in these cases? It is not ordinarily esteemed a wise act to dissipate a fortune, and the wisdom is not more manifest because the intention is pious. What, for example, should we think of a man who, having a substantial sum left in his gift for the service of the poor, turned it into pence, and gave to everybody who pleaded hunger the price of a bun?

Yet this is precisely what many executors have been doing of late. They would seem to have aimed at nothing so much as the effacement of the splendid offerings committed to their disposal, and although we readily admit that the position is not without its difficulties, and that to carefully weigh the numerous appeals—to parry the importunities of friends, and finally to make and hold fast to a choice, which embraces but a few of many applicants, and satisfies nobody but those who are chosen, perhaps not all of them, requires not a little patience and some moral courage, we should say that anyone who feels himself to be unendowed with a full measure of these qualities had better renounce the task he has been allotted.

But assuming that executors will continue to shrink from a course which they consider invidious—though why it should be so in their case more than in that of a testator is hard to say—and who ever heard of a testator who subdivided his gifts among the whole race of charities or relations?—then, would it not be better, as it would certainly be more logical, to ensure participation to the whole body of accredited institutions, though not by a division of capital?

Let us suppose that one-half of the funds we have referred to, and which have now vanished in air, had been invested for the benefit of the hospitals, there would have been an annual income arising almost sufficient to double in perpetuity the present grants of the Hospital Sunday Fund. It will be a step of no little importance towards the solution of the chronic hospital difficulty when it is recognised that large and exceptional benefactions are better dealt with as a whole, or at least by division into portions substantial enough to render them enduring. Agricola.

